# Characteristics of type I diabetes patients during first visit at Hiperdia Minas Center, Viçosa, MG

**DOI:** 10.1186/1758-5996-7-S1-A190

**Published:** 2015-11-11

**Authors:** Daniela Neves Ribeiro, Bruna Soares Faria, Flávia Xavier Valente, Milene Cristine Pessoa

**Affiliations:** 1 Centro Hiperdia Minas Viçosa, Viçosa, Brazil

## Background

The Hiperdia Minas Center in Viçosa aims to attend patients with chronic diseases such as hypertension, cardiovascular disease, diabetes mellitus and chronic kidney disease, referred by the Primary Healthcare Assistance. Thus, it is important to know the type 1 diabetes patients' profile of this Center in order to promote self-care actions.

## Objective

Describe the profile of all type 1 diabetes patients first attended by the Hiperdia multidisciplinary team.

## Materials and methods

All type 1 diabetes patients attended by the Hiperdia Minas Center were enrolled at the study, except for pregnant women. The data were collected from medical records at the first attending. At this first visit, the patients were attended by a physician, nutritionist, pharmacist, nurse, psychologist, physiotherapist, and social worker. Socioeconomics, anthropometrics, biochemistry and clinic data were assessed. Qualitative data were described as absolute and relative frequency, and the quantitative data as median, minimum, and maximum values.

## Results

At the time of the study, 72 type 1 diabetes patients had the first visit at the Center. Out of these 54.2% were women. The minimum age was 3 months old, and the maximum 57 yrs. old. The median age was 20 yrs. old. Regarding nutritional status, 8.3% of the patients were obese; 22.2% were overweight or at risk of overweight; 65.3% were normal weight, and 4.2% were low weight. The median diagnosis time was 4 yrs.; the maximum time was 30 yrs., and minimum 6 days. The majority of the patients reported being single (72.2%), having income below 1 minimum wage (37.5%), and less than 12 yrs. of study (84.8%). Also, the majority of patients is not alcoholic or smoker, and almost 50% did not practice physical activity. Regarding insulin type, the majority of the patients were using NPH or NPH + regular insulin (88.9%), did not have comorbidities (84.7%) and showed glycated hemoglobin above 7% (87.5%).

## Conclusion

It was observed that type 1 diabetes patients attended at the Hiperdia Minas Center are heterogeneous, showing the need to adapt the self-care promotion actions according to the patients' characteristics in order to favor more adhesion to the treatment.

